# Facile Preparation of Highly Conductive Poly(amide-imide) Composite Films beyond 1000 S m^−1^ through Ternary Blend Strategy

**DOI:** 10.3390/polym11030546

**Published:** 2019-03-22

**Authors:** Yanbin Wang, Huang Yu, Yongchao Li, Teng Wang, Tao Xu, Jinxing Chen, Zicheng Fan, Yufeng Wang, Biaobing Wang

**Affiliations:** Jiangsu Key Laboratory of Environmentally Friendly Polymeric Materials, School of Materials Science and Engineering, Jiangsu Collaborative Innovation Center of Photovolatic Science and Engineering, Changzhou University, Changzhou 213164, China; wangyanbin@cczu.edu.cn (Y.W.); 15051960012@163.com (H.Y.); 15261179872@163.com (Y.L.); 18351220755@163.com (T.W.); ba1486436lia@163.com (T.X.); 15161123052@163.com (J.C.); 15365931925@163.com (Z.F.); 18306116537@163.com (Y.W.)

**Keywords:** conductive polymer composite, ternary blend, poly(amide-imide), polyaniline, carbon black

## Abstract

Highly conductive thin films with suitable mechanical performances play a significant role in modern electronic industry. Herein, a series of ternary conductive polymer composites were fabricated by incorporating carbon black (CB) into binary conductive polymer composites of poly(amide-imide) (PAI) and polyaniline (PANI) to enhance their mechanical and conductive properties simultaneously. By varying the composition of PAI/PANI/CB ternary films, the conductivity enhanced by two orders of magnitude compared with the sum of PAI/PANI and PAI/CB binary conductive polymer composites, and a high conductivity of 1160 S m^−1^ was achieved. The improved conductivity is mainly because much more continuous conductive networks were constructed in the ternary conductive polymer composites. With the help of the unusual morphology, the tensile strength was also enhanced by more than 80% from 21 to 38 MPa. The origin for the improved morphology was discussed for further improvement.

## 1. Introduction

Conductive polymer composites (CPCs) have been attracting more and more attention in modern electronic industry due to their unique advantages such as flexibility, light weight, and high-throughput processing for large-area devices [1,2,3,4,5,6]. For most of the polymeric materials in our life, their conductivities are very low because a cluster of free electrons could not flow along their backbones. For example, poly(amide-imides) (PAIs) possessing good mechanical and thermal properties have been considered to be excellent engineering materials, but their application in the conductive field has been limited due to their poor conductivity [7,8,9,10,11]. The insulator–conductor transition could be achieved by incorporating conductive fillers into the polymer matrix. Usually, organic conductive fillers are classified into two groups—intrinsically conductive polymers (ICPs) [12,13,14,15,16,17,18] and carbonaceous fillers (CFs) [19,20,21,22,23,24,25]. Polyaniline (PANI), an intrinsically conductive polymers, has been considered one of the most important conducting polymers to fabricate CPCs, because of its low-cost, relative environmental stability, and electrochemical behavior [26,27,28,29,30]. However, the conductivity of most CPCs based on this kind of conductive filler is relatively low [31,32,33]; more importantly, their mechanical performance clearly deteriorates with the increasing content of ICPs [17,34,35]. Carbon nanotubes (CNTs), as a kind of carbonaceous filler, have been regarded as ideal conductive fillers, which can improve conductive and mechanical performance simultaneously [36,37,38,39,40]. Unfortunately, the high cost of this kind of conductive filler makes them unusable in large-scale commercial use. Furthermore, these carbonaceous fillers are prone to aggregate in a polymer matrix because (a) there is strong inter-tube van der Waals interaction in the carbonaceous fillers and (b) the interfacial interaction between the carbonaceous fillers and the polymer is very weak. Therefore, it is worth developing a facile method to fabricate CPCs with highly conductive, suitable mechanical properties.

Recently, ternary conductive polymer composites (TCPCs) have been developed as a simple and effective strategy to enhance their mechanical properties and reduce their cost [41,42,43,44,45,46,47,48,49,50]. More specifically, TCPCs are classified into three types in terms of conductive fillers: ICPs–ICPs, ICPs–CFs, and CFs–CFs. For ternary blends based on ICPs–ICPs and CFs–CFs, they suffer from their parent disadvantages such as poor mechanical performance and high cost. On the other hand, for ternary blends based on ICPs–CFs, they combine the advantages of ICPs (low cost) and CFs (high mechanical performance) through suitable design. Indeed, functionalized multi-walled carbon nanotubes (FMWCNTs) were added into PAI and PANI binary composites to prepare PAI/PANI/FMWCNT ternary composites [41]. It was found that the conductive fillers of FMWCNTs and PANI dispersed in the PAI matrix very well due to the hydrogen bond interaction among PAI, FMWCNTs, and PANI. Thus their tensile strength was enhanced by 40%. More interestingly, it was found that there is a synergistic effect of PANI and FMWCNTs on the conductivity, and their conductivities increased obviously from 10^−3^ to 8.3 S m^−1^. It is a pity that the conductivity is still not enough high for applications that require high conductivity. It has been reported that the interaction between PANI and carbon black (CB) is much more impressive due to its smaller particle size (larger surface area), and lower particle density (higher particle porosity) [51]. Thus, it is believed that the combination of CB and PANI is a much more effective pairing to fabricate TCPCs.

Herein, a simple process is used to prepare highly conductive polymer composites by incorporating CB into PAI/ANI binary composites. The manufacturing process is achieved by the one-step solvent-casting method followed by a drying process. By varying the composition of PAI/PANI/CB ternary composites, their conductive and mechanical properties were researched. Moreover, the tensile strength was enhanced by more than 80%, from 21 to 38 MPa, and a high conductivity of over 1000 S m^−1^ was achieved, which is one of the highest values for the TCPCs based on intrinsically conductive polymers and carbonaceous fillers [52,53,54]. The evolution of the morphology as a function of CB content was also researched to elucidate the origin of the improvement in electrical and mechanical performance.

## 2. Materials and Methods

### 2.1. Materials

Carbon black (VXC72) was obtained from Cabot Corporation (Boston, MA, USA). Ammonium persulfate, Camphorsulfonic acid, 1,4-dihydroxybenzene, 1,4-diaminobenzene, pyridine, *N*-methyl-2-pyrrolidone, 4,4′-oxy-diphthalic anhydride, and aniline were provided by Tokyo Chemical Industry (Shanghai, China). Calcium chloride, 11-aminoundecanoic acid, lithium chloride, and triphenyl phosphite were provided by Sigma-Aldrich (Shanghai, China). Other materials were provided by Aladdin Industrial Corporation (Shanghai, China).

### 2.2. Synthesis of Aromatic PAI

**Figure 1:**
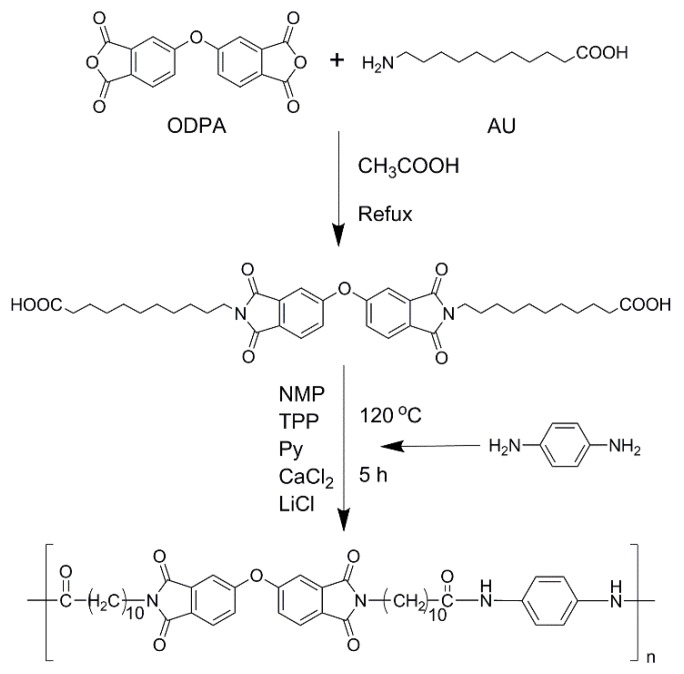
Synthetic route of diimide diacid monomer and aromatic poly(amide-imide) (PAI).

**Figure 2:**
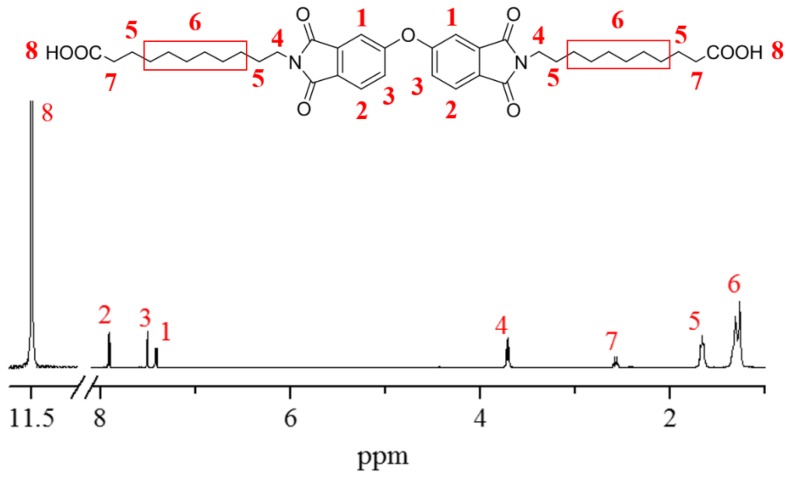
H-NMR spectrum of diimide diacid monomer.

**Figure 3:**
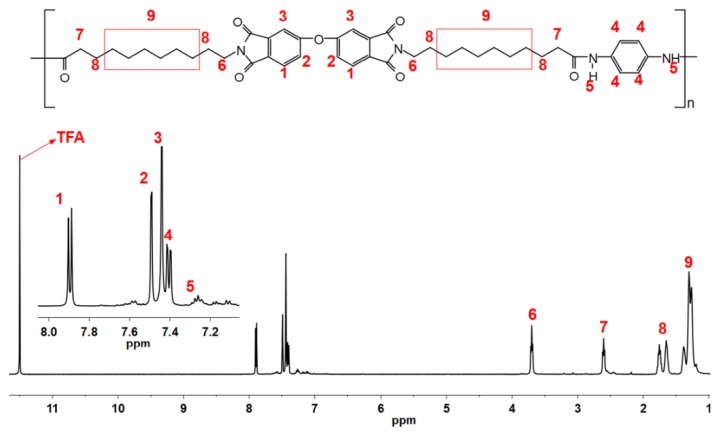
H-NMR spectrum of poly(amide-imide).

As shown in Figure 1, aromatic PAI was prepared from diimide diacid and diamine through a Yamazaki–Higashi phosphorylation reaction [7,17], and their structures were confirmed by H-NMR as shown in Figure 2 and Figure 3. 

**Preparation of diimide diacid monomer (DIDA):** The mixture of 4,4′-oxy-diphthalic anhydride (ODPA) (6.20 g, 0.02 mmol), 11-aminoundecanoic acid (AU) (8.04 g, 0.04 mmol), and acetic acid (CH_3_COOH) (35 mL) was first agitated for 12 h at 30 °C, and then heated slowly to reflux for another 10 h in a nitrogen atmosphere. The mixture was cooled to room temperature, dropped into acetic acid, and then filtered. Finally, the crude product was washed by deionized water three times, and dried in a vacuum box at 50 °C overnight (yield: 71%). ^1^H-NMR (δ/ppm, d-trifluoroacetic acid): 11.50 (s, 2H), 7.91 (d, 2H), 7.52 (d, 2H), 7.42 (dd, 2H), 3.72 (t, 4H), 2.43 (t, 4H),1.67 (m, 8H), 1.29 (m, 24H). FTIR (KBr, ν, cm^−1^): 3467, 1771, 1694, 1393, 1366, 1265, 1079, 745.

**Preparation of aromatic PAI:** The mixture of DIDA (6.7 g, 0.01 mol), 1,4-diaminobenzene (1.1 g, 0.01 mol), calcium chloride (4 g, 0.036 mol), lithium chloride (2 g, 0.048 mol), triphenyl phosphite (10 mL), and *N*-methyl-2-pyrrolidone (20 mL) was heated slowly to reflux overnight, then cooled to room temperature. The reactants were poured into methanol and filtered. The precipitate was washed by warm deionized water and methanol sequentially, and dried in a vacuum box at 60 °C for 8 h. Finally, aromatic PAI with an inherent viscosity of 1.86 dL g^−1^ was obtained (yield: 92%). ^1^H-NMR (δ/ppm, d-trifluoroacetic acid): 7.88 (d, 2H), 7.47 (s, 2H), 7.41 (s, 2H), 7.38 (d, 4H), 3.68 (t, 4H), 2.67 (t, 4H), 1.72 (m, 8H), 1.23 (m, 24H). FTIR (KBr, ν, cm^−1^): 3294, 1652, 1547, 1514.

### 2.3. Preparation of PANI

**Synthesis of PANI:** The mixture of freshly purified aniline (3.72 g, 40 mmol) and 30 mL of 0.2 M HCl was stirred for 0.5 h at 0 °C. Then ammonium persulfate (11.12 g, 40 mmol) dissolved in 60 mL of water was slowly dropped into the aniline solution. The color of the mixture became dark green gradually, and then the mixture was continuously agitated for another 12 h so that the crude product could settle down. PANI was filtered, then washed with NH_4_OH solution and deionized water alternately until the filtrate became neutral. Low-molecular-weight PANI was removed by a Soxhlet extractor (washing solvent: acetone and dichloromethane), and the final product exhibited an inherent viscosity of 0.35 dL g^−1^. 

**Doping of PANI:** First, PANI (200 mg) and camphorsulfonic acid (120 mg) were placed into *m*-cresol (66 g), and the mixture was stirred for 12 h, dropped onto a glass substrate, then moved into an oven and dried at 80 °C until the *m*-cresol evaporated completely. 

### 2.4. Preparation of PAI/PANI/CB Ternary Conductive Polymer Composites

The PAI/PANI/CB ternary mixture was prepared with compositions of PAI:PANI:CB = 40:8:*x* mg mL^−1^ (0 ≤ *x* ≤6.5) in *m*-cresol. Firstly, the suspension was sonicated for 15 min at 30 °C, then dropped onto glass substrates and dried at 80 °C until *m*-cresol evaporated completely.

### 2.5. Characterization

Fourier transform infrared spectroscopy (FTIR) spectra of samples were measured by an Avatar 370 spectrometer (Thermo Nicolet, Madison, WI, USA), and the data were recorded in the region of 4000–500 cm^−1^ with a resolution of 0.1 cm^−1^. Proton nuclear magnetic resonance (^1^H-NMR) spectra of samples were investigated by Avance III 400M NMR (PerkinEimer, Waltham, MA, USA) using trifluoroacetic acid (TFA) as the solvent and tetramethylsilane as the internal reference. Thermal stability of binary and ternary composites was researched by a Perkin-Elmer TGA 4000 (Waltham, MA, USA) under a heating rate of 10 °C/min in a nitrogen atmosphere. The glass transitions of TCPCs samples were investigated by differential scanning calorimetry (DSC) (Perkin-Elmer, DSC8000) in a nitrogen atmosphere from −29 to 300 °C under a heating speed of 30 °C/min. The instrument was calibrated for both heat flow and temperature using indium and zinc standards. The thicknesses of films were measured by micrometer caliper (Mitutoyo, CLM1-15QM, Kawasaki, Japan) with a resolution of 0.1 μm. The conductivities of PAI/PANI/CB composites were recorded by a standard four-point probe method on RTS-9 at room temperature; four samples were prepared and investigated for each concentration of carbon black, and the conductivity of each sample was evaluated from the average of six measurements at different positions. The tensile strength of samples was tested by universal testing measurement (Kaiqiangli, WDT-5, Shenzhen, China) at a strain rate of 50 mm min^−1^ at room temperature; the samples were prepared with a size of 10 mm × 70 mm. The morphology of samples was investigated by field emission scanning electron microscopy (SUPRA, 12–1000000×, Zeiss, Jena, Germany). 

## 3. Results

### 3.1. Electrical Conductivity

**Figure 4:**
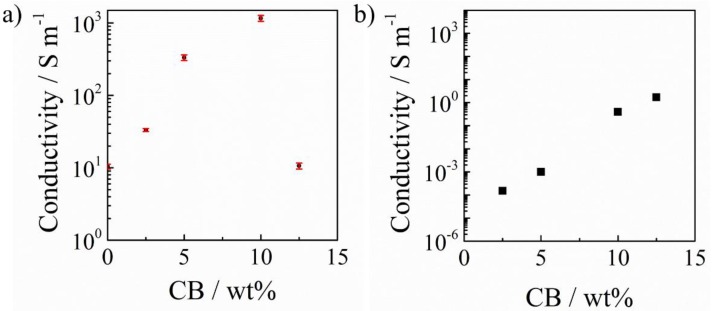
Variation of electronic conductivity as a function of carbon black (CB) weight ratio in the PAI/polyaniline (PANI)/CB ternary (**a**) and PAI/CB binary (**b**) conductive polymer composites.

Previously, we fabricated a series of PAI/PANI conductive polymer composites [17], and the conductive property of PAI/PANI polymer composites was clearly improved compared with pure PAI. A conductivity of 10 S m^−1^ was achieved when the content of PANI reached 20 wt%, but this was still low. To further increase the conductivity of PAI composites, we incorporated CB into PAI/PANI binary blend composites to fabricate PAI/PANI/CB ternary blend composites. The in-line four-point probe method was adopted to measure the conductivity of PAI/PANI binary and PAI/PANI/CB ternary films, and their conductivities were obtained according to Equation (1):

(1) $$\sigma_{\mathrm{dc}}=1/\left(4.532\times R\times l\right)$$

where *σ*_dc_ is the DC conductivity, *R* is the sheet resistance, 4.532 is the correction factor, and *l* is the thickness of the films [55]. In general, with adding CB into PAI/PANI binary films, the conductivity of PAI/PANI/CB ternary films increased hugely. In detail, PAI/PANI binary blend composites exhibited a conductivity of 9.6 S m^−1^, which is almost the same as what we reported before [17]. As shown in Figure 4a, the conductivity of PAI/PANI/CB ternary films increased exponentially. Excitingly, the conductivity of ternary films increased to 1160 S m^−1^ when the content of CB reached 10 wt%. On the other hand, for the PAI/CB binary films, although their conductivity was clearly enhanced by increasing the content of CB, as shown in Figure 4b, it was still limited to 1.7 S m^−1^ even when the content of CB reached as high as 12.5 wt%. In other words, the conductivity of PAI/PANI/CB ternary films is somewhat higher than that of the sum of PAI/PANI binary and PAI/CB binary films, indicating that there is an impressive synergistic effect of PANI and CB in the conductivity. However, when adding more CB, the conductivity of PAI/PANI/CB ternary films decreased, probably because the conductive filler aggregated in the PAI matrix, as discussed later. Furthermore, the swelling behavior of the conductive films was investigated. It was found that with adding CB into the PAI/PANI binary blend, its water absorption decreased slightly (2.8% for PAI/PANI binary film, 1.9%, 1.3%, 1.7%, and 1.9% for PAI/PANI/CB ternary films with 2.5 wt%, 5 wt%, 10 wt%, and 12.5 wt% CB). This indicated that the conductive property of PAI/PANI/CB ternary composites is less affected by water compared with PAI/PANI binary composites.

### 3.2. Tensile Strength

**Figure 5:**
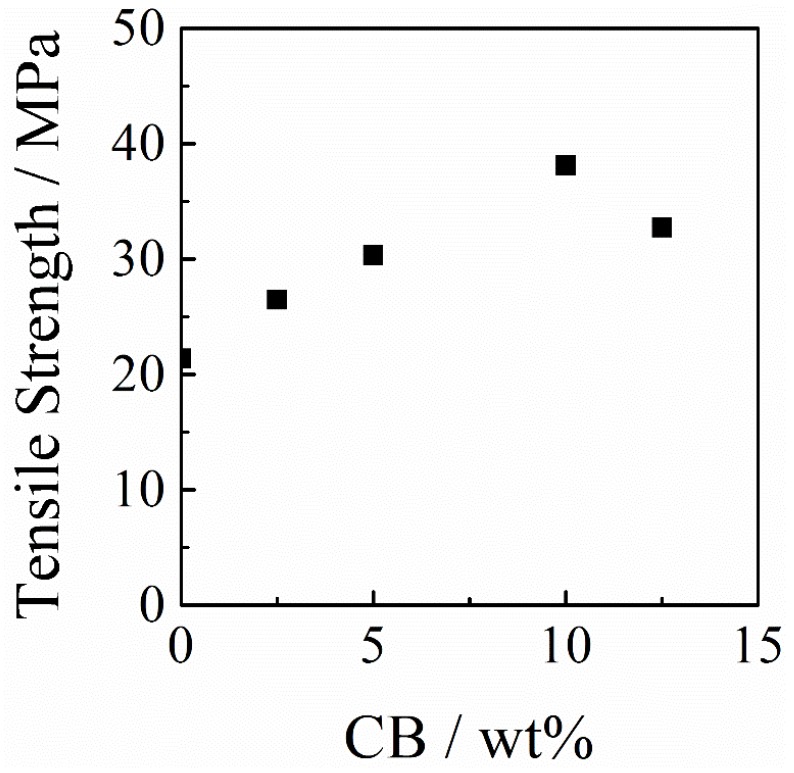
Variation of tensile strength as a function of the CB weight ratio in the PAI/PANI/CB ternary blend films.

CB has been not only considered as conductive filler, but also as a good reinforcing agent [56,57,58]. Figure 5 shows the effect of the CB weight ratio on the tensile strength of PAI/PANI/CB ternary blend films. It was found that the tensile strength of PAI/PANI/CB ternary blend films improved linearly with increasing content of CB. When the weight ratio of CB reached 10 wt%, the tensile strength improved by 80%, from 21 to 38 MPa. The enhancement of mechanical performance probably can be attributed to the improved morphology, and discussed later. By further increasing the content of CB to 12.5 wt%, the tensile strength decreased slightly. The change in the trend of mechanical properties coincided with the change in the trend of conductivity, as mentioned before. 

### 3.3. Thermal Stability

**Figure 6:**
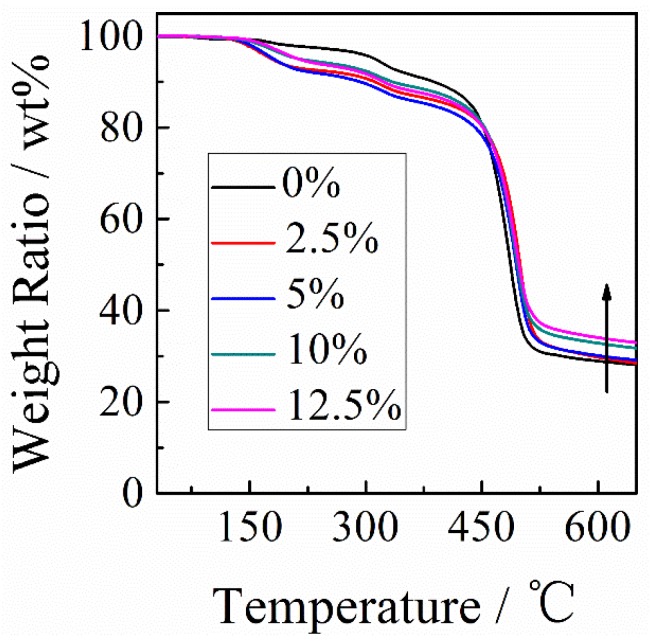
Thermogravimetric analysis curves of PAI/PANI/CB ternary composites with different compositions.

Figure 6 shows the thermogravimetric analysis curves of PAI/PANI/CB ternary composites with different compositions. It was found that both PAI/PANI binary and PAI/PANI/CB ternary composites showed three distinct decomposition stages. In detail, the first decomposition process in the temperature range of 120–290 °C is attributed to the removal of the doping molecule (camphorsulfonic acid). The second decomposition stage observed between 270 and 330 °C mainly results from the degradation of the PANI backbone. The third decomposition stage above 450 °C mainly results from the degradation of PANI and PAI chains. From Table 1, it can be seen that the thermal stability of PAI/PANI/CB ternary composites improved slightly with increasing content of CB. Furthermore, higher char yields were obtained because of the more thermally stable aromatic structure of CB. 

On the other hand, the limiting oxygen index (LOI) can be calculated from char yield (*Y*_c_) in accordance with the van Krevelen–Hoftyzer equation [59].

(2) LOI=17.5+0.4 CR

where *CR* is the char yield in wt% at 700 °C. As summarized in Table 1, the LOI of PAI/PANI/CB ternary composites was raised with increasing content of CB. Moreover, the LOIs of PAI/PANI binary and PAI/PANI/CB ternary composites were much higher than 21%, indicating that all investigated samples can be considered as self-extinguishing materials.

### 3.4. Phase Transition

**Figure 7:**
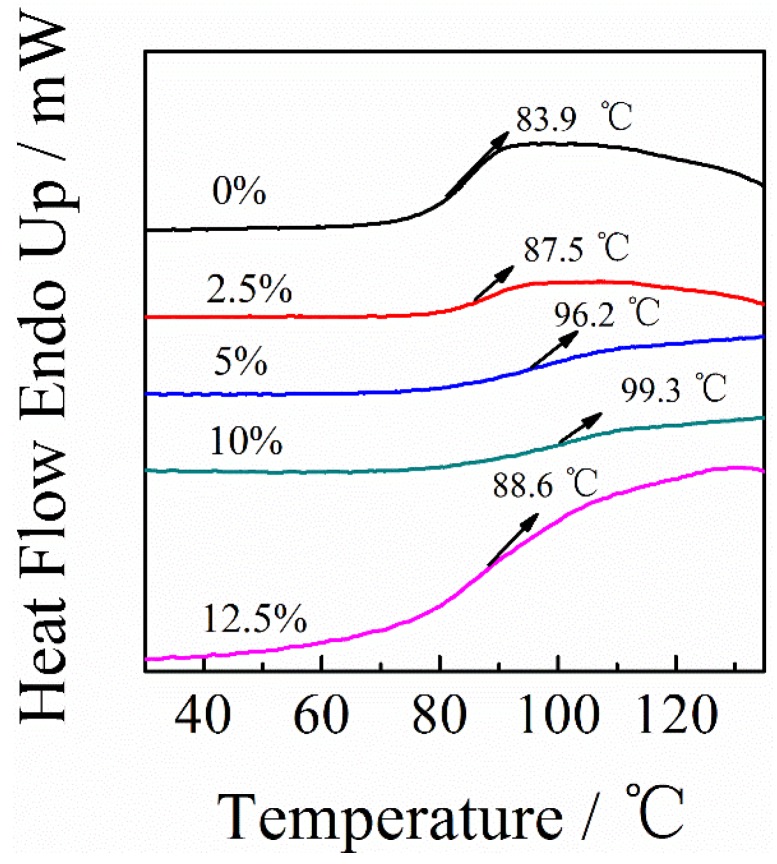
Differential scanning calorimeter (DSC) analysis curves of PAI/PANI/CB ternary composites with different weight ratios of CB.

The phase transition behavior of PAI/PANI/CB ternary blend composites with different weight ratios of CB was investigated by differential scanning calorimetry (DSC) at a heating speed of 30 °C/min in a nitrogen atmosphere. As shown in Figure 7, by increasing the content of CB to 10 wt%, the glass transition temperature (*T*_g_) of PAI/PANI/CB ternary blend composites increased from 83.9 to 99.3 °C, suggesting that hydrogen bonding interactions exist between PAI/PANI and CB. By further increasing the content of CB to 12.5 wt%, the *T*_g_ decreased slightly, which indicated that the hydrogen bonding interaction between PAI/PANI and CB was weakened, probably because CB aggregated in the PAI/PANI matrix. 

## 4. Discussion

As mentioned above, the mechanical and conductive performances of PAI/PANI/CB ternary blend composites were noticeably improved compared with those of PAI/PANI binary blend composites. There are two main reasons for this: (1) the weight ratio of CB in the ternary blend composites and (2) the morphology evolution of the blend films.

**Figure 8:**
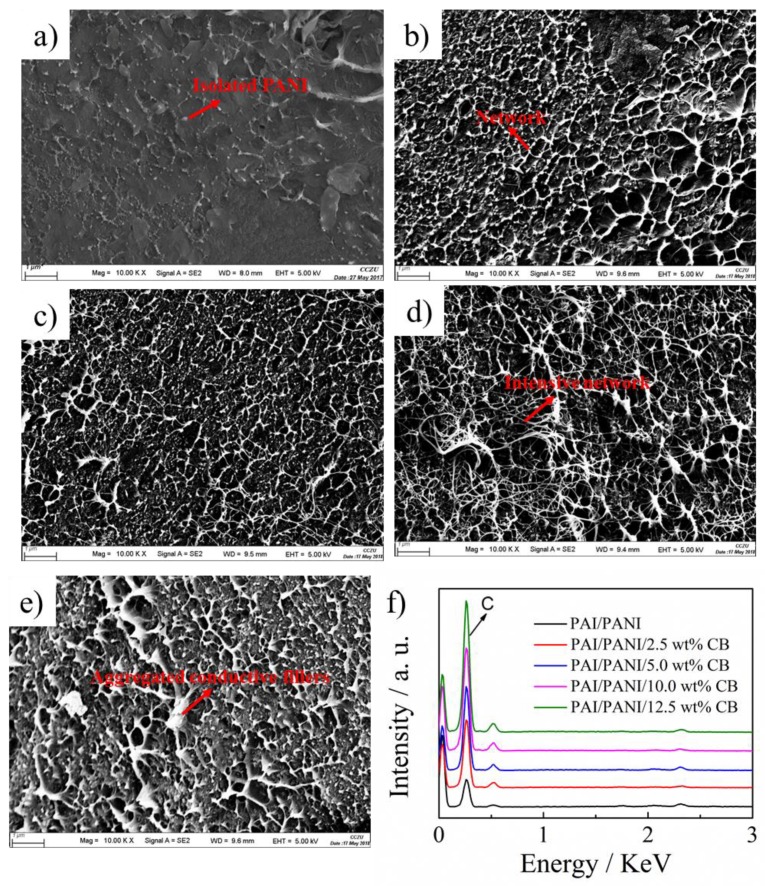
Scanning electron microscopy images of PAI/PANI binary (**a**) and PAI/PANI/ functionalized multi-walled carbon nanotube (FMWCNT) ternary composites with 2.5 wt% (**b**), 5 wt% (**c**), 10 wt% (**d**), and 12.5 wt% (**e**) CB. Energy dispersive X-ray analysis of conductive films (**f**).

In order to disclose the origin of the improved properties, the dispersion and morphology of PAI/PANI/CB ternary blend composites with different weight ratios of CB were investigated by field emission scanning electron microscopy (FESEM). As shown in Figure 8, PANI exhibited a node fibril morphology in the PAI/PANI binary blend composite, and was surrounded by a PAI matrix. In other words, most of the PANI conductive filler was discontinuous in the PAI/PANI binary blend composite. By adding CB into the PAI/PANI binary blend composite, as expected, the carbon content increased with the increasing weight ratio of CB, as shown Figure 6f. Furthermore, the isolated conductive fillers were connected to a network, which is beneficial for electron flow and stress spread. Thus, CB plays the role of a bridge to construct the percolating network. The conductivity and tensile strength increased from 9.6 to 33.3 S m^−1^ and from 21 to 26 MPa, respectively. By further increasing the content of CB to 10 wt%, the conductive network became more intensive but without large-scale aggregation, and thus the conductivity further increased to 1160 S m^−1^. A more intensive network means many more molecular chains carry the inner stress in the ternary blend, and the homogeneous stress distribution resulted in an obvious enhancement of mechanical properties [57], thus the tensile strength further increased to 38 MPa. Moreover, it should be noted that the conductivity of PAI/PANI/CB ternary films was remarkably higher than that of the sum of PAI/PANI and PAI/CB binary films, suggesting there is a great synergistic effect of PANI and CB on the conductivity. When the content of CB was further increased to 12.5 wt%, conductive fillers aggregated in the PAI matrix, and the conductive and mechanical properties deteriorated slightly. Therefore, it can be concluded that the blend composition and morphology play key roles in the conductive and mechanical performances of the blend.

**Figure 9:**
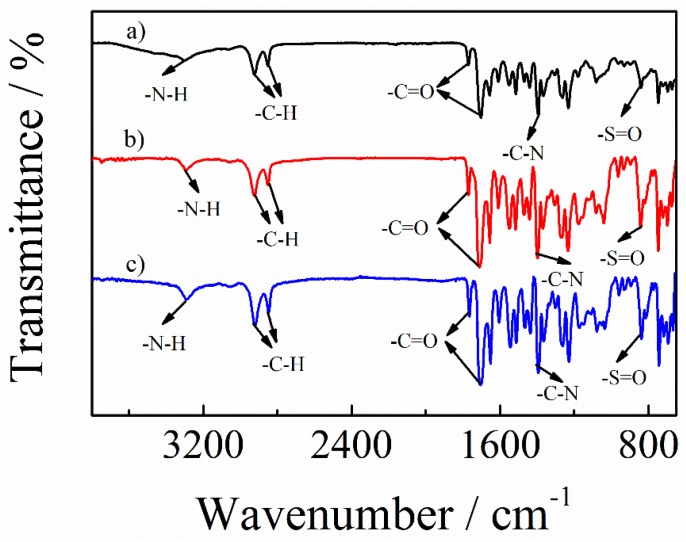
Fourier transform infrared spectroscopy (FTIR) spectra of PAI (black), PAI/PANI binary (red line), and PAI/PANI/CB ternary composite with a weight ratio of 10 wt% CB (blue line).

Now, we discuss the possible interaction between PAI, PANI, and CB. Although there is a π–π interaction between carbonaceous fillers and the polymer matrix, carbonaceous fillers are still prone to cluster in the polymer matrix. For the purpose of improving the dispersion of conductive fillers, a much stronger intermolecular force should be adopted. As mentioned before, the *T*_g_ of PAI/PANI/CB ternary blend composites increased when adding CB into the PAI/PANI binary composite, probably because the amide group of PAI and polar groups of CB exhibited a hydrogen bonding interaction, which led to a template effect, imparting an enhanced thermal stability to the PAI/PANI/CB ternary blend composites [41]. The hydrogen bonding interaction among PAI, PANI, and CB could be confirmed again by the chemical shift of the FTIR spectra, as shown in Figure 9. For the spectrum of PAI neat film, the bands at 1769 and 1706 cm^−1^ are the symmetric and asymmetric stretching of carbonyl groups. The presence of peaks around 1368, 1067, and 746 cm^−1^ corresponded to the vibration of the imide heterocyclic ring. The band at 3301 cm^−1^ is ascribed to N–H bending of the amide group. With adding PANI into PAI matrix, the N–H bending of the amide group was blue shifted to 3296 cm^−1^, suggesting a hydrogen bonding effect existing between PAI and PANI. For the spectrum of PAI/PANI/CB ternary blend composites, the N–H vibration of the amide group was further blue shifted to 3289 cm^−1^, indicating that the hydrogen bonding interaction of polar groups in the ternary blend composites was strengthened. It is believed that the hydrogen bonding interaction in the PAI/PANI/CB ternary blend composites is helpful in breaking the intermolecular force in the CB and enhancing the interfacial adhesion among PAI, PANI, and CB, which is beneficial for the improvement in conductive and mechanical performances [60].

Finally, we discuss the future direction for highly conductive TCPCs based on intrinsically conductive polymers and carbonaceous fillers. It is well known that the conductivity (1/resistivity) of composites can be expressed by a scaling law on the basis of classical percolation theory [61]:

(3) $$\sigma =\sigma_{o}{\left(p-p_{c}\right)}^{t}$$

where *p*_c_ is the percolation threshold of the CPCs, *p* is the weight ratio of conductive filler in the CPCs, *σ*_o_ is a scaling factor, *σ* is the conductivity of the CPCs, and *t* is an exponent that is related to the dimensionality of the conductive network within CPCs. In models, *t* ≈ 1.3 and *t* ≈ 2.0 are used for two- and three-dimensional networks, respectively. From Equation (3), it is obvious that the conductivity of TCPCs could be further improved through controlling the morphology from 2D to 3D. Indeed, there are several reports that enhance the conductivity of TCPCs through constructing 3D structures using 2D graphene and 1D carbon nanotubes [62,63,64]. Recently, Lu prepared a 3D hierarchical structure of a PANI–graphene hybrid by combining 1D PANI nanorods with 2D graphene nanosheets [65]. Therefore, it is believed that the pairing of 1D PANI and 2D graphene is a much more impressive combination to improve the conductivity of TCPCs through careful morphology design.

## 5. Conclusions

In summary, a series of ternary conductive polymer composites have been fabricated by incorporating CB into a PAI/PANI binary blend. The conductivity of PAI/PANI/CB ternary blend composites was improved hugely, and a high conductivity of over 1000 S m^−1^ was achieved. The improved conductive property is mainly a result of the more intensive conductive network formed in the ternary blend, which is also useful in stress spread. As a result, the tensile strength increased by 80% from 21 to 38 MPa. We believe that this finding is generally applicable to other ternary conductive composites. Furthermore, we propose that a much higher conductivity of TCPCs could be obtained by using 1D intrinsically conductive polymers and 2D graphene to construct a 3D conductive network. 

## Figures and Tables

**Table 1:** Thermal properties of PAI/PANI/CB ternary blends with different compositions.

Composition	First Stage		Second Stage		Third Stage		*T*_g_ °C	*Y*_c_ %	LOI %
	*T*_o_/°C	*T*_max_/°C	*T*_o_/°C	*T*_max_/°C	*T*_o_/°C	*T*_max_/°C			
0%	119	147	273	300	453	485	83.9	27	28
2.5%	128	164	295	321	472	499	87.5	28	29
5%	136	172	281	324	464	493	96.2	28	29
10%	156	175	292	321	463	492	99.3	31	30
12.5%	154	188	291	321	464	494	88.6	32	30

*T*_o_ is decomposition temperature at 5% loss; *T*_max_ is maximum decomposition temperature; the value of the limiting oxygen index (LOI) is calculated from *Y*_c_ at 700 °C.
